# Genome-wide identification and expression profiling of the *NHX* gene family in oat (*Avena sativa* L.)

**DOI:** 10.1186/s12864-026-12519-y

**Published:** 2026-01-29

**Authors:** Yuqi Li, Meifeng Liu, Wenjie Zhao, Feng Yuan, Zhenyu Jia, Yi Wang, Wendong Zheng, Chengchen Pan, Chunxiang Fu

**Affiliations:** 1 Synthetic Biology Technology Innovation Center of Shandong Province, Qingdao Institute of Bioenergy and Bioprocess Technology, Chinese Academy of Sciences, Qingdao, China; 2 Shandong Energy Institute, Qingdao, China; 3 Inner Mongolia Mengcao Seed Industry Science & Technology Research Institute Co., Ltd., Hohhot, China; 4 Mengcao Ecological Environment (Group) Co., Ltd., Hohhot, China; 5 Qingdao Haier Biological Medical Technology Co., Ltd., Qingdao, China

**Keywords:** *Avena sativa*, Salt stress, *NHX* gene family, qRT-PCR

## Abstract

**Background:**

Salt stress imposes osmotic imbalance and ion toxicity, severely impairing crop growth and development. Na⁺/H⁺ antiporters (NHXs) play a central role in salt tolerance by mediating sodium transport across cellular membranes.

**Results:**

We conducted a genome-wide characterization of the *NHX* gene family in four hexaploid oat cultivars, identifying 126 *NHX* genes. These were grouped into 33 orthologous gene groups (OGGs), comprising 10 core and 23 dispensable OGGs. The naked oat cultivar ‘Sanfensan (SFS)’ contained the largest number of both total and core *NHX* genes. Phylogenetic analysis classified *AsNHX* genes into three classes: vacuolar (Vac-class), endosomal (Endo-class), and plasma membrane (PM-class). Ka/Ks analysis indicated strong purifying selection across most members. *Cis*-regulatory element analysis revealed abundant stress-related motifs, including abscisic acid (ABA) and methyl jasmonate (MeJA) response elements, suggesting roles in abiotic stress adaptation. Transcriptome and qRT-PCR data confirmed that *AsNHX1/3/7/9/14/23/24/25/32* were up-regulated under salt stress, highlighting their importance in salinity tolerance.

**Conclusions:**

This study provides the first comprehensive characterization of the *AsNHX* gene family, detailing their evolutionary, structural, and functional features. The findings offer critical insights into salt adaptation mechanisms in oat and identify promising targets for genetic improvement of salinity tolerance.

**Supplementary Information:**

The online version contains supplementary material available at 10.1186/s12864-026-12519-y.

## Background

Soil salinization is a major abiotic constraint that threatens global agriculture and sustainable crop production. Approximately 800 million hectares of farmland are affected by salinity, with saline-alkali soils in China alone representing ~ 20% of the cultivated area [1–3]. Excess sodium accumulation disrupts photosynthesis, nutrient balance, and cellular integrity, leading to sharp yield losses across crop species [4]. Climate change and poor irrigation practices further intensify soil salinization, underscoring the urgent need to develop salt-tolerant varieties [5].

Plants employ multiple strategies to mitigate salt stress, with ionic homeostasis being a central factor in tolerance [6]. The conserved SALT OVERLY SENSITIVE (SOS) signaling pathway regulates sodium extrusion and sequestration through calcium-mediated signaling [7, 8]. *NHX* antiporters act as critical executors of this pathway, mediating Na⁺/H⁺ exchange to restore ion balance [9]. *NHXs* function in coordination with SOS pathway components, particularly CBL-CIPK complexes, to regulate sodium compartmentalization and exclusion [10]. While other adaptive mechanisms, such as osmolyte synthesis (e.g., proline, glycine betaine) and antioxidant activity (SOD, CAT, POD), contribute to salt tolerance, these processes ultimately depend on the establishment of ionic equilibrium, highlighting the central role of NHX transporters [11].

As members of the CPA1 family of cation/proton antiporters, NHXs facilitate electroneutral exchange of protons with sodium or potassium ions across membranes [12]. Based on subcellular localization and function, they are classified into Vac-class, Endo-class, and PM-class transporters [13–15]. Functional studies have established their significance in salt tolerance. For example, heterologous expression of *AtNHX1* enhanced vacuolar sodium sequestration and salinity tolerance in transgenic plants [16]. In cotton, *GbNHX2* expression was strongly induced by salt stress, contributing to ion balance [17]. Similarly, overexpression of *OsNHX1* enabled plants to withstand 100 mM NaCl stress through enhanced sodium compartmentalization [18]. Collectively, these findings demonstrate the utility of *NHX* genes as targets for engineering salinity resilience.

Oat (*Avena sativa* L.), particularly the naked oat cultivar ‘Sanfensan (SFS)’, is an important hexaploid cereal cultivated globally in temperate regions [19, 20]. Its nutritional richness—β-glucans, proteins, and essential lipids—combined with adaptability to marginal soils, makes it valuable for both food and feed [21, 22]. Oats can thrive where other cereals fail, positioning them as promising candidates for cultivation in salinity-affected regions [23, 24]. Despite their agronomic significance, the *NHX* gene family in oat remains poorly studied compared to *Arabidopsis* (8 members), rice (7 members), wheat (30 members), and cotton (23–24 members) [25–28]. Key questions concerning their genome-wide distribution, duplication, evolutionary trajectories, and stress-related functions remain largely unexplored.

To bridge this gap, we performed the first systematic analysis of the *NHX* gene family in four representative oat cultivars, including SFS. The objectives were to: (1) identify *NHX* genes genome-wide and characterize their physicochemical features; (2) assess evolutionary relationships and duplication events via phylogenetic and synteny analyses; (3) evaluate expression dynamics and cis-regulatory elements under salt stress; and (4) predict protein–protein interaction networks to reveal regulatory roles. This study provides a comprehensive resource for dissecting *NHX*-mediated salt adaptation in oat and identifies genetic targets to guide marker-assisted breeding for enhanced salinity tolerance in cereal crops.

## Materials and methods

### Plant materials and salt stress treatment

The naked oat (*Avena sativa* var. *nuda*) cultivar ‘Huazao-2’ was used in this study. Uniform seeds were germinated at 24 °C under a 16-hour light/8-hour dark photoperiod. Seedlings at the two-leaf stage were transferred to hydroponic culture with Hoagland’s nutrient solution. At the three-leaf stage, plants were exposed to salt stress by adding 100 mM NaCl to the nutrient solution. Leaf samples were collected at 0, 2, 4, 8, 12, and 24 h post-treatment, immediately frozen in liquid nitrogen, and stored at − 80 °C for RNA extraction. Each treatment included three biological replicates, with three seedlings pooled per replicate.

### Identification of the *AsNHX* gene family

Genomic data for four oat cultivars— ‘Marvellous’, ‘OT3098v2’, ‘Sang’, and ‘Sanfensan (SFS)’—were obtained from the OatBioDB database (http://waoOat.cn/). ‘Marvellous’, ‘OT3098v2’, and ‘Sang’ are hulled oat cultivars, while ‘SFS’ is a naked oat. Genomic data for *Oryza sativa*, *Triticum aestivum*, *Arabidopsis thaliana*, *Sorghum bicolor*, and *Hordeum vulgare* were retrieved from Ensembl Plants (http://plants.ensembl.org/index.html). The Hidden Markov Model (HMM) profile for the NHX domain (PF00999) was downloaded from Pfam (https://pfam.xfam.org) and searched against oat genomes using HMMER 3.0 [29] with an E-value cutoff of 1e^− 5^. In parallel, BLAST searches were conducted in TBtools [30] using known NHX protein sequences from rice, wheat, Arabidopsis, sorghum, and barley. Candidates identified by both approaches (E-value < 1e^− 5^) were retained as putative *NHX* genes. All candidates were further validated using the Conserved Domain Database (CDD) (https://www.ncbi.nlm.nih.gov/cdd/). TBtools was used to calculate physicochemical properties of AsNHX proteins, including molecular weight and isoelectric point.

### Phylogenetic, gene Structure, conserved Domain, and motif analyses

NHX protein sequences from the four oat cultivars and five reference species were aligned using ClustalW, and a phylogenetic tree was constructed in MEGA 7.0 with the Maximum Likelihood method and 1,000 bootstrap replicates [31]. The tree was visualized in iTOL [32]. Gene structures were determined using genome annotations. Conserved motifs were predicted with MEME (https://meme-suite.org/meme/) [33], and conserved domains were confirmed using the NCBI CDD database. (https://www.ncbi.nlm.nih.gov/cdd/). All results were visualized using TBtools [30]. Motif enrichment analysis was conducted using the Analysis of Motif Enrichment (AME) tool from MEME Suite v5.5.8. Statistical significance was evaluated with Fisher’s exact test, followed by Benjamini-Hochberg correction for multiple testing. Motifs with adjusted *P*-values < 0.05 were considered significantly enriched.

### *Cis*-Regulatory element analysis

For each *AsNHX* gene, a 2.0-kb sequence upstream of the translation initiation codon (ATG) was defined as the promoter region. *Cis*-acting regulatory elements were predicted using PlantCARE (http://bioinformatics.psb.ugent.be/webtools/plantcare/html/). Identified CREs were classified into functional categories (e.g., stress-responsive, hormone-responsive, light-responsive) and visualized as clustered heatmaps in TBtools [30].

### Chromosomal localization and synteny analysis

The chromosomal positions of *AsNHX* genes were determined with TBtools using genome annotation files [30]. Synteny analysis was performed with MCScanX [34] to examine collinearity within the four oat genomes and between ‘SFS’ and other oat cultivars (‘Marvellous’, ‘OT3098v2’, ‘Sang’) as well as rice, wheat, sorghum, and barley. Ks (synonymous), Ka (nonsynonymous), and Ka/Ks ratios were calculated in TBtools for all homologous gene pairs [30].

### Protein-Protein interaction (PPI) analysis

The PPI network of AsNHX proteins in ‘SFS’ was predicted using STRING (https://cn.string-db.org/), with *Arabidopsis* proteins as the reference model [35]. The network was visualized in Cytoscape 3.10.0 [36].

### Transcriptome data analysis

Transcriptome datasets related to salt stress in oat were obtained from the NCBI Sequence Read Archive (BioProject accession PRJNA355375) (https://www.ncbi.nlm.nih.gov/sra/?term=Oat). The oat variety ‘HanYou-5’ was grown in Hoagland nutrient solution until the three-leaf stage and subsequently treated with 100 mM NaCl. Plant samples were collected at 2, 4, 8, 12, and 24 h post-treatment for transcriptome sequencing. Expression levels of *NHX* genes were quantified as log₂(TPM + 1), and expression patterns were visualized as heatmaps using TBtools [30].

### Quantitative Real-Time PCR (qRT-PCR)

Total RNA was extracted using the RNAprep Pure Plant Kit (Tiangen, Beijing, China). First-strand cDNA was synthesized with the PrimeScript RT Reagent Kit (TaKaRa, Dalian, China). Gene-specific primers are listed in Table S7. *AsACTIN* was used as the internal control. qRT-PCR reactions were performed with the SYBR Green Pro Taq HS kit following the manufacturer’s protocol, using a Roche LightCycler 96 instrument (Roche, Basel, Switzerland). Amplification was conducted with a three-step PCR cycling program. Three biological replicates were analyzed for each sample. Relative expression levels were calculated using the 2^⁻ΔΔCT^ method [37]. Statistical significance was assessed using Student’s *t*-test.

### Subcellular localization

NHX-eGFP was introduced into *Agrobacterium* GV3101 and grown overnight at 28 °C in LB medium. Cells were collected and resuspended in infiltration buffer (10 mM MgCl_2_; 10 mM MES-KOH, pH 5.5; 100 µM acetosyringone) to OD_600_ = 0.8, then pressure-infiltrated into leaves of 4-week-old *Nicotiana benthamiana* plants. After 24 h darkness, plants were transferred to standard conditions (16 h light/8 h dark) for 48 h, GFP was visualized using an Olympus FV3000 confocal microscope. Primer sequences are listed in Table S7.

## Results

### Identification, Phylogeny, and physicochemical properties of *NHX* genes

**Fig. 1:**
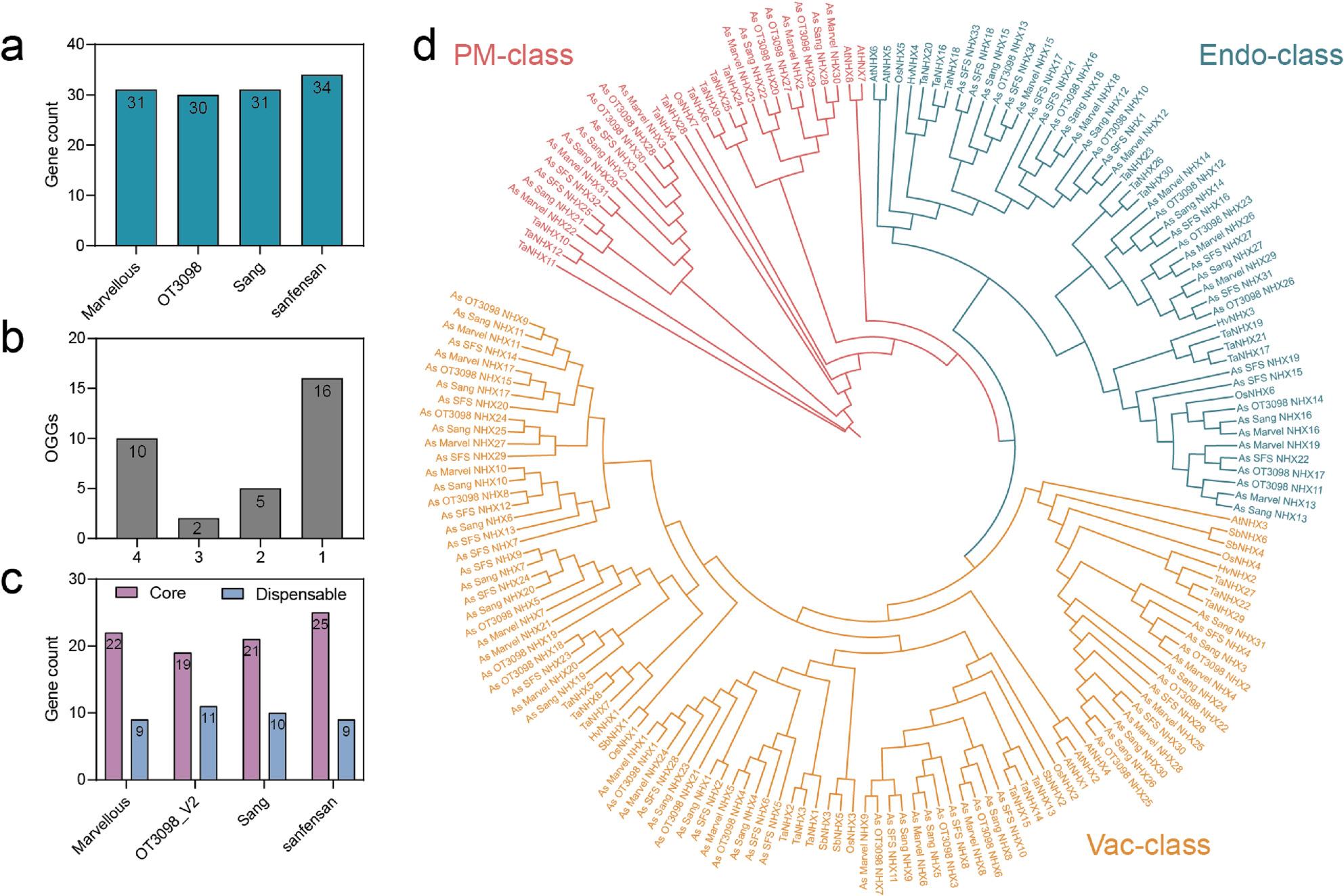
Identification and phylogenetic analysis of NHX genes in four oat genomes. **a** Genomic distribution of NHX genes across four oat genome assemblies. **b** Core and dispensable orthogroups (OGGs). The x-axis indicates the number of genomes in which an OGG is present. OGGs shared by all four genomes are designated as core, while those present in three, two, or one genome(s) are classified as dispensable. **c** Distribution of core and dispensable NHX genes among the four genomes. **d** Phylogenetic relationships of NHX proteins from four oat cultivars (Avena sativa, As), Oryza sativa (Os), Arabidopsis thaliana (At), Triticum aestivum (Ta), Hordeum vulgare (Hv), and Sorghum bicolor (Sb). Proteins cluster into three distinct groups, each highlighted in a different color

Using HMMER and BLASTP, 31, 30, 31, and 34 *NHX* family genes were identified in the oat cultivars ‘Marvellous’, ‘OT3098’, ‘Sang’, and ‘SFS’, respectively (Fig. 1a). The NHX proteins were systematically named according to chromosomal positions (Marvellous: *As_Marvel_NHX1-31*; OT3098: *As_OT3098_NHX1-30*; Sang: *As_Sang_NHX1-31*; SFS: *As_SFS_NHX1-34*) (Table S1). Physicochemical analysis revealed wide variation in sequence length, molecular weight, and isoelectric point (pI). In ‘SFS’, proteins ranged from 99 to 1138 aa, with pI values from 4.91 (*As_SFS_NHX17*) to 10.51 (*As_SFS_NHX13*), and molecular weights of 10.92 kDa (*As_SFS_NHX33*) to 126.29 kDa (*As_SFS_NHX3*). ‘Sang’ proteins spanned 153–1137 aa, with pI values of 5.13 (*As_Sang_NHX15*) to 9.07 (*As_Sang_NHX26*), and molecular weights of 16.55 kDa (*As_Sang_NHX30*) to 126.10 kDa (*As_Sang_NHX2*). In ‘OT3098’, proteins were 204–1137 aa, with pI values from 4.71 (*As_OT3098_NHX10*) to 9.13 (*As_OT3098_NHX3*), and molecular weights between 22.16 kDa (*As_OT3098_NHX29*) and 126.07 kDa (*As_OT3098_NHX30*). ‘Marvellous’ proteins ranged from 121–1137 aa, with pI values from 5.13 (*As_Marvel_NHX15*) to 8.69 (*As_Marvel_NHX1*), and molecular weights of 13.25 kDa (*As_Marvel_NHX30*) to 126.08 kDa (*As_Marvel_NHX31*). These findings highlight marked diversity in NHX family members across cultivars (Table S2).

To assess conservation in the oat pan-genome, *NHX* genes were clustered into orthologous gene groups (OGGs) using a 95% amino acid similarity cutoff. The 126 identified genes formed 33 OGGs (Fig. 1b, Table S3). This total exceeded that of any individual genome, reflecting presence/absence variations (PAVs). Of the 33 OGGs, 10 were core *NHX* genes present in all cultivars, while 23 were dispensable (absent in at least one). The dispensable set included 2 OGGs shared by three cultivars, 5 shared by two, and 16 unique to single cultivars. ‘SFS’ contained the most core genes (25), whereas ‘OT3098’ had the fewest (19). In contrast, ‘OT3098’ carried the largest number of dispensable genes (11), while ‘Marvellous’ and ‘SFS’ each contained only 9 (Fig. 1c).

A phylogenetic tree of 181 *NHX* proteins from rice (*Oryza sativa*), wheat (*Triticum aestivum*), Arabidopsis (*Arabidopsis thaliana*), sorghum (*Sorghum bicolor*), barley (*Hordeum vulgare*), and the four oat cultivars confirmed close evolutionary relationships between oat and wheat *NHX* genes (Fig. 1d). Consistent with findings in *Arabidopsis*, the sequences grouped into three classes: vacuolar (Vac-class), predicted to localize to vacuoles; endosomal (Endo-class), localized to endosomal compartments; and plasma membrane (PM-class), associated with the plasma membrane. The Vac-class was the largest, comprising 70 oat proteins, followed by the Endo-class (37 proteins) and the PM-class (19 proteins).

### Chromosomal localization and collinearity analysis of *AsNHX* genes

**Fig. 2:**
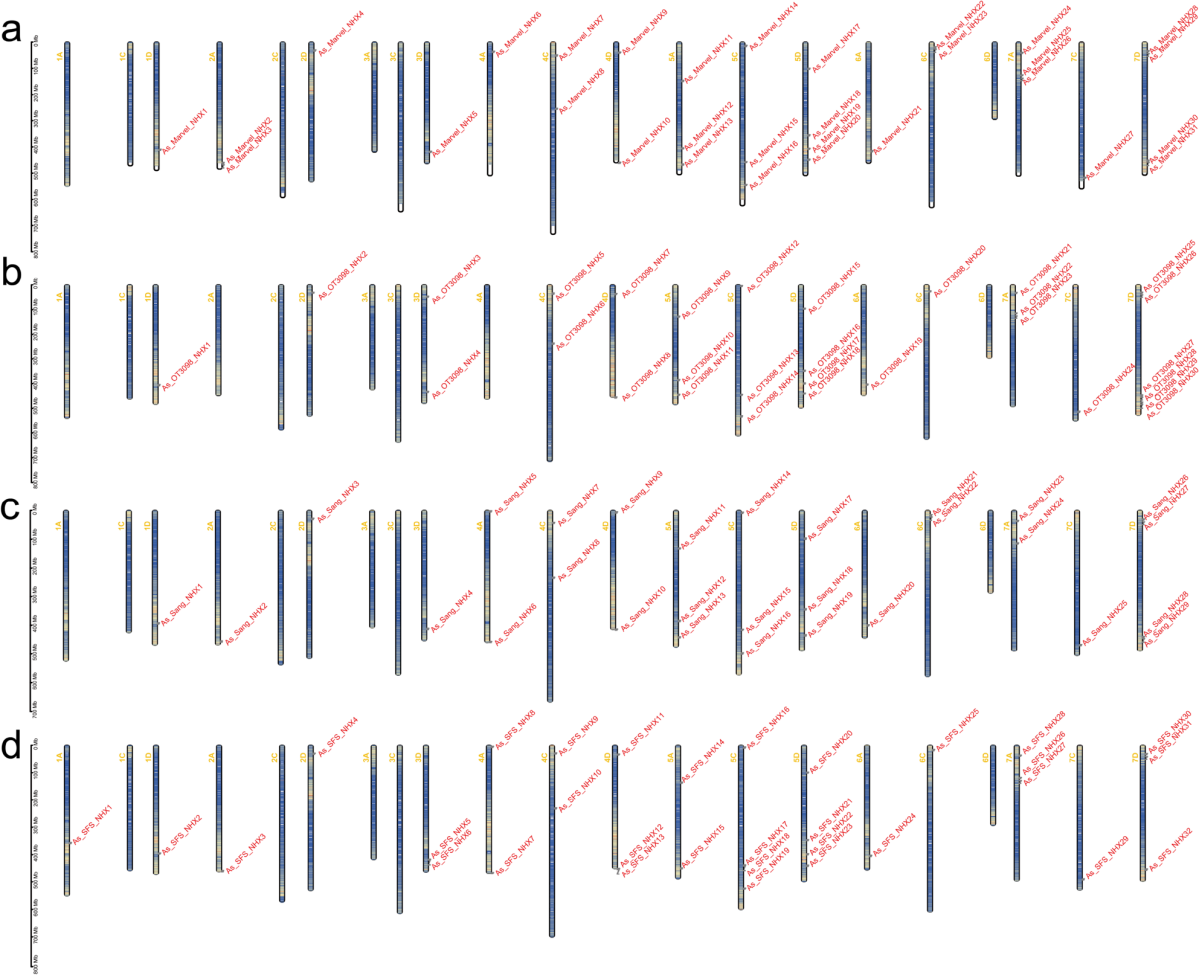
Chromosomal distribution of the NHX gene family in ‘Marvellous’ (**a**), ‘OT3098’ (**b**), ‘Sang’ (**c**), and ‘SFS’ (**d**). Yellow labels (left) indicate chromosome numbers, and red labels (right) indicate gene IDs

Chromosomal mapping revealed broad but uneven distribution of *AsNHX* genes (Fig. 2). In ‘Marvellous’, 31 genes were located on 15 chromosomes, with 10, 8, and 13 genes in the A, C, and D subgenomes, respectively (Fig. 2a). ‘OT3098’ carried 30 genes across 12 chromosomes (7, 7, and 16 in A, C, and D subgenomes) (Fig. 2b). In ‘SFS’, 34 genes were distributed across 16 chromosomes, with 10, 8, and 14 in the A, C, and D subgenomes, plus 2 on unplaced scaffolds (Un) (Fig. 2c). ‘Sang’ contained 31 genes on 15 chromosomes, with 9, 8, and 12 in the A, C, and D subgenomes, plus 2 on Un (Fig. 2d). Overall, *AsNHX* genes were enriched in the D subgenome. Notably, chromosomes 1 C, 2C, 3 A, 3 C, and 6D lacked *NHX* genes in all four cultivars.

**Fig. 3:**
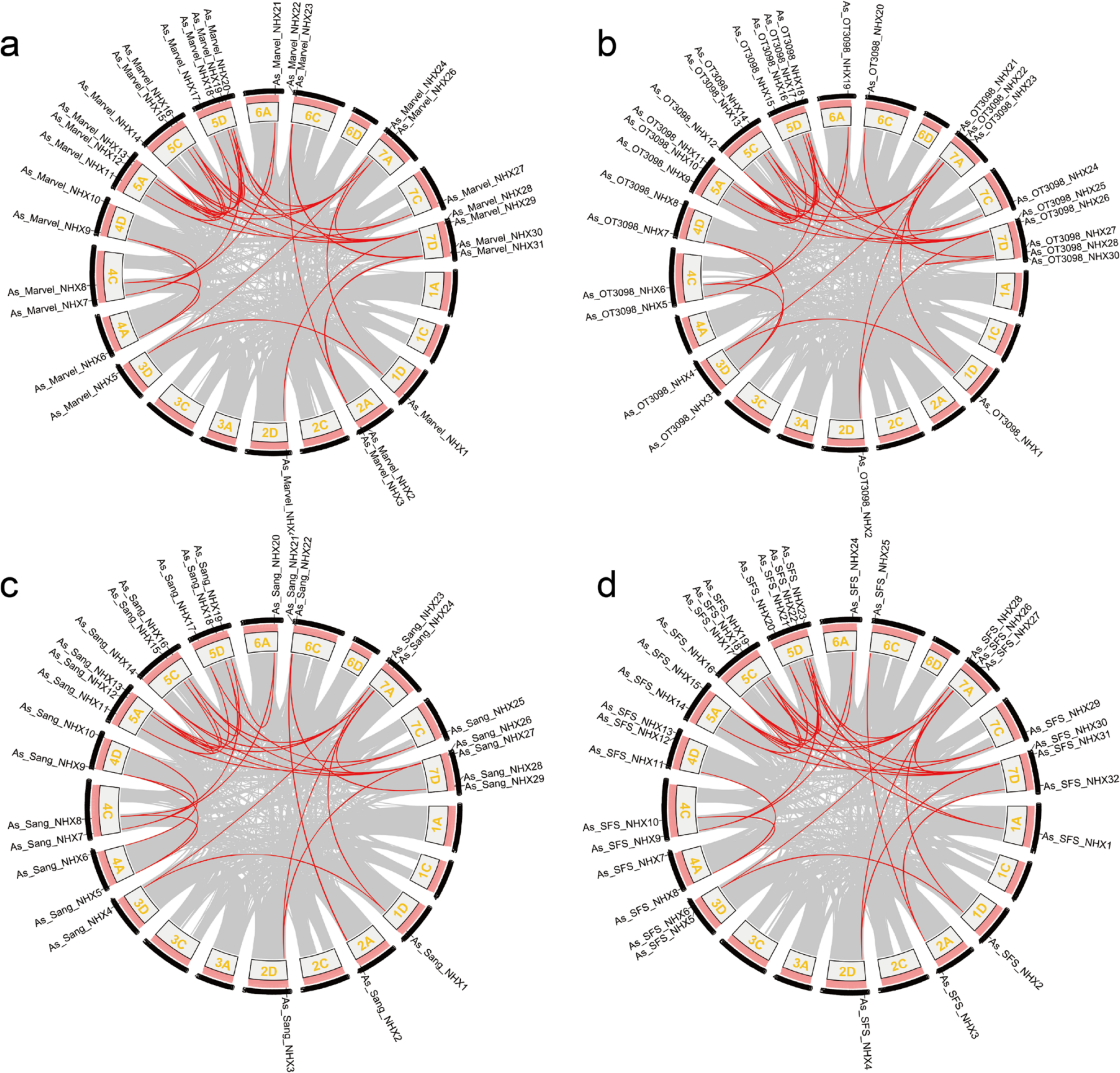
Collinearity of NHX genes in ‘Marvellous’ (**a**), ‘OT3098’ (**b**), ‘Sang’ (**c**), and ‘SFS’ (**d**). Syntenic gene pairs are connected by red lines

Gene duplication analysis identified 36, 34, 30, and 34 homologous gene pairs in ‘Marvellous’, ‘OT3098’, ‘Sang’, and ‘SFS’, respectively (Fig. 3). Evolutionary pressures were assessed using Ka/Ks ratios (Ka/Ks > 1: positive selection; =1: neutral; <1: purifying selection) (Table S4). Eight gene pairs—*As_Marvel_NHX11/27*, *As_Marvel_NHX17/27*, *As_OT3098_NHX9/24*, *As_OT3098_NHX15/24*, *As_Sang_NHX11/25*, *As_Sang_NHX17/25*, *As_SFS_NHX14/29*, and *As_SFS_NHX20/29*—displayed Ka/Ks > 1, indicating adaptive evolution. All others had Ka/Ks between 0 and 1, consistent with purifying selection.

**Fig. 4:**
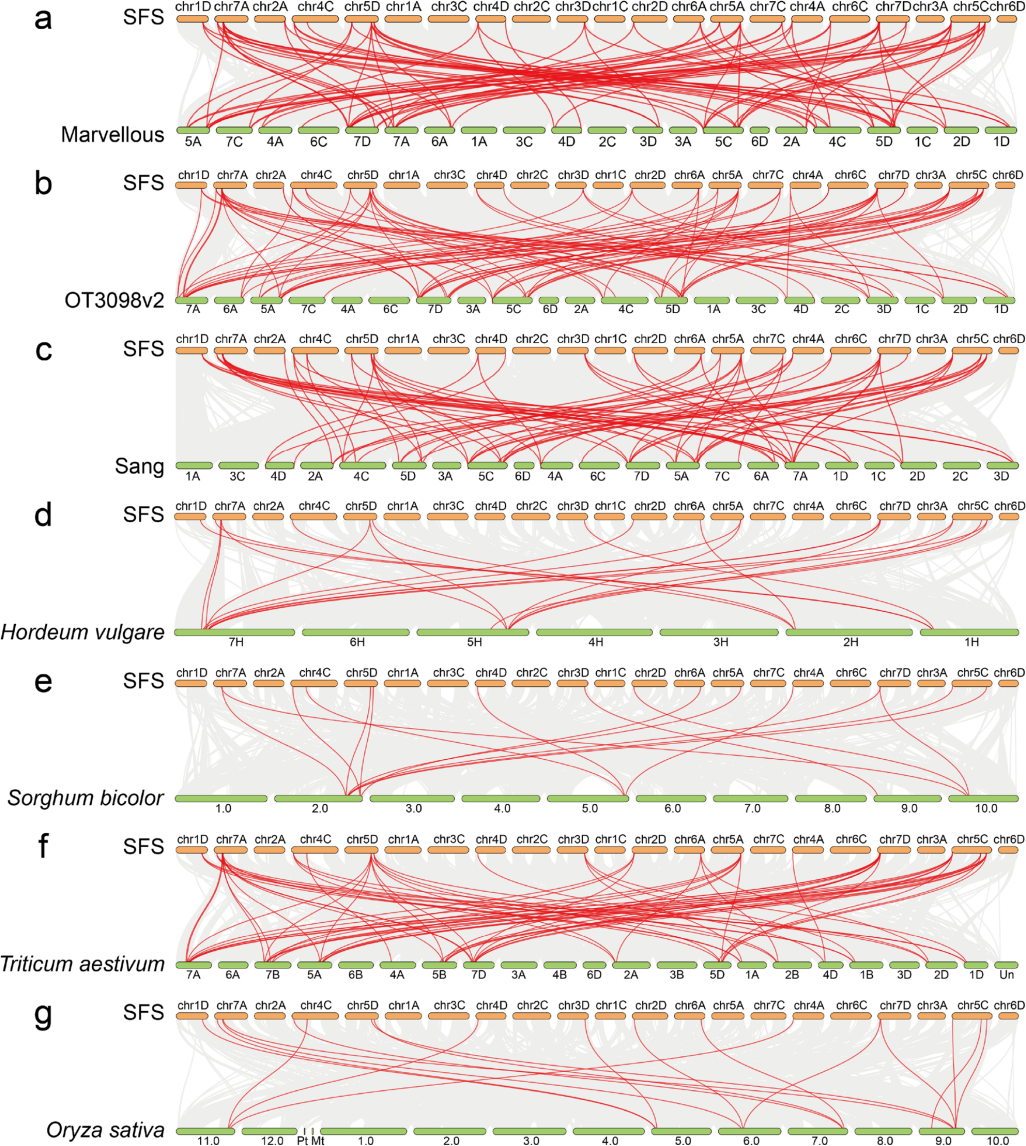
Comparative collinearity of NHX genes between naked oat ‘SFS’ and ‘Marvellous’ (**a**), ‘OT3098’ (**b**), ‘Sang’ (**c**), Hordeum vulgare (**d**), Sorghum bicolor (**e**), Triticum aestivum (**f**), and Oryza sativa (**g**). Gray lines indicate all collinear blocks; red lines highlight collinear NHX gene pairs

Collinearity analysis of naked oat ‘SFS’ with ‘Marvellous’, ‘OT3098’, ‘Sang’, *Hordeum vulgare*, *Sorghum bicolor*, *Triticum aestivum*, and *Oryza sativa* revealed extensive synteny (Fig. 4). ‘SFS’ shared 87 collinear pairs with ‘Marvellous’ (Fig. 4a), 86 with ‘OT3098’ (Fig. 4b), and 74 with ‘Sang’ (Fig. 4c). Cross-species comparison identified 22 collinear pairs with *Hordeum vulgare* (Fig. 4d), 16 with *Sorghum bicolor* (Fig. 4e), and 18 with *Oryza sativa* (Fig. 4g). The strongest homology was observed with *Triticum aestivum*, with 69 collinear pairs (Fig. 4f), consistent with phylogenetic clustering. These results suggest that orthologous genes, frequently shaped by duplication events, have contributed to oat evolution and expansion of the *AsNHX* family.

### Genome-Wide phylogenetic assessment of oat cultivars

To clarify phylogenetic relationships among the four oat cultivars, we performed a genome-wide species tree analysis using OrthoFinder. Contrary to initial inferences based on synteny pair counts, the species tree revealed a distinct topology: (SFS, (Sang, (Marvellous, OT3098))) (Fig. S1). This indicates that ‘SFS’ represents the earliest diverging lineage, followed by ‘Sang’, with ‘Marvellous’ and ‘OT3098’ forming the most closely related clade (bootstrap support = 0.70). These genome-wide phylogenetic results confirm the greater evolutionary divergence of naked oat ‘SFS’ from the hulled cultivars ‘Sang’, ‘Marvellous’, and ‘OT3098’.

### Analysis of conserved Motifs, Domains, and gene structure of *NHX* genes

**Fig. 5:**
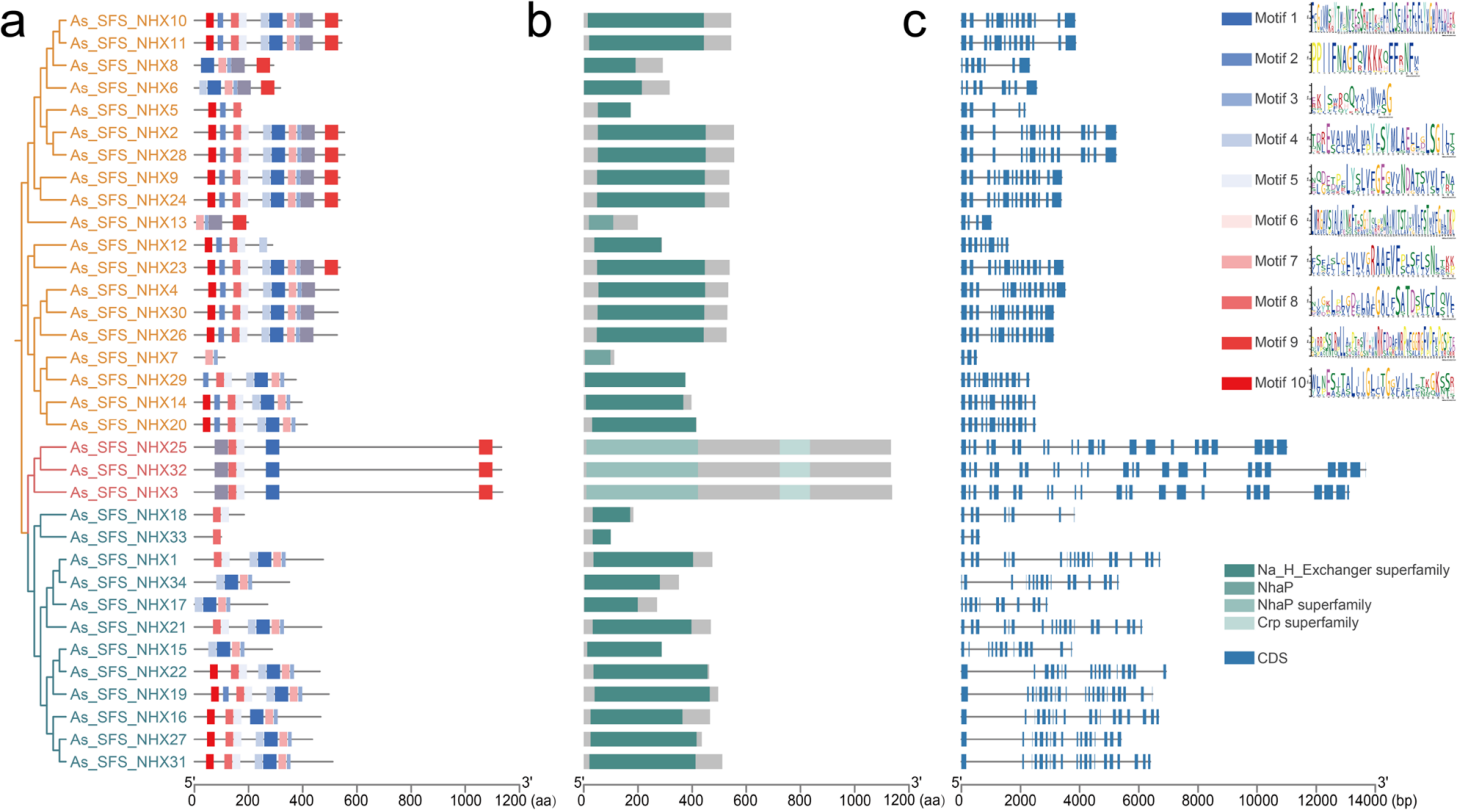
Motifs, conserved domains, and gene structures of NHX genes in ‘SFS’. **a** Top 10 conserved motifs of NHX proteins. The sequence logo for each motif is adjacent to the map. **b** Distribution of conserved domains. **c** Gene structures, with exons in blue boxes and introns in gray lines. Scale bars in (**a**) and (**b**) indicate amino acid length, while the scale in (**c**) represents nucleotide sequence length. Proteins are ordered according to a maximum-likelihood phylogenetic tree, with subfamilies color-coded as in Fig. 1

To explore the structural diversity of *NHX* genes in ‘SFS’, conserved motifs were analyzed using MEME, identifying 10 distinct motifs across their protein sequences (Fig. 5a). The sequence logos and detailed consensus sequences for each motif are provided in Fig. 5a and Table S5. PM-class *NHX* genes contained motifs 1, 5, 6, 8, and 9. Most Endo-class members carried motifs 1, 3, 4, 5, 7, and 8, while *As_SFS_NHX33* contained only motif 8. Vac-class genes encompassed all 10 motifs. While motif composition and number varied significantly between classes, they were highly conserved within each class, supporting the classification system and indicating structural conservation among *NHX* proteins. The strict conservation of specific motif combinations within each class strongly supports the phylogenetic classification and suggests divergent functional specialization among *NHX* subfamilies.

Motif enrichment analysis using AME quantified the relative abundance and significance of identified motifs. Motifs 7 and 3 were the most significantly enriched, present in 79.41% of NHX sequences but only 0.02% of whole-genome sequences, representing an approximate 4,000-fold enrichment (Table S6). Their conservation across the majority of NHX members indicates that these motifs encode critical functional domains potentially involved in transmembrane transport, ion binding, or protein–protein interactions typical of NHX exchangers. Notably, the combinatorial presence of multiple enriched motifs within individual NHX sequences suggests a modular architecture, where conserved regions may cooperate to mediate diverse functional roles during plant development and stress responses.

Domain analysis showed that, in addition to the Na_H_Exchanger superfamily domain, *As_SFS_NHX13* and *As_SFS_NHX7* harbored the NhaP domain, implicating them in K⁺/H⁺ transmembrane exchange (Fig. 5b). Moreover, PM-class members contained both NhaP and Crp superfamily domains. The Crp family is known to sense intracellular cAMP levels and contributes to salt stress responses. These results suggest that PM-class members play central roles in maintaining ion homeostasis and regulating salt stress adaptation in naked oat.

Gene structure analysis revealed marked variability in exon–intron organization (Fig. 5c). Intron numbers ranged from 2 to 23. PM-class genes *NHX3*, *NHX25*, and *NHX32* contained the most introns (22–23). The Endo-class averaged 14.25 introns per gene, whereas the Vac-class carried the fewest, averaging 10.11 introns. Differences in intron number among the three classes likely contribute to gene length variation and further support structural conservation within oat *NHX* genes.

### Analysis of *Cis*-Acting elements in *NHX* promoter regions

**Fig. 6:**
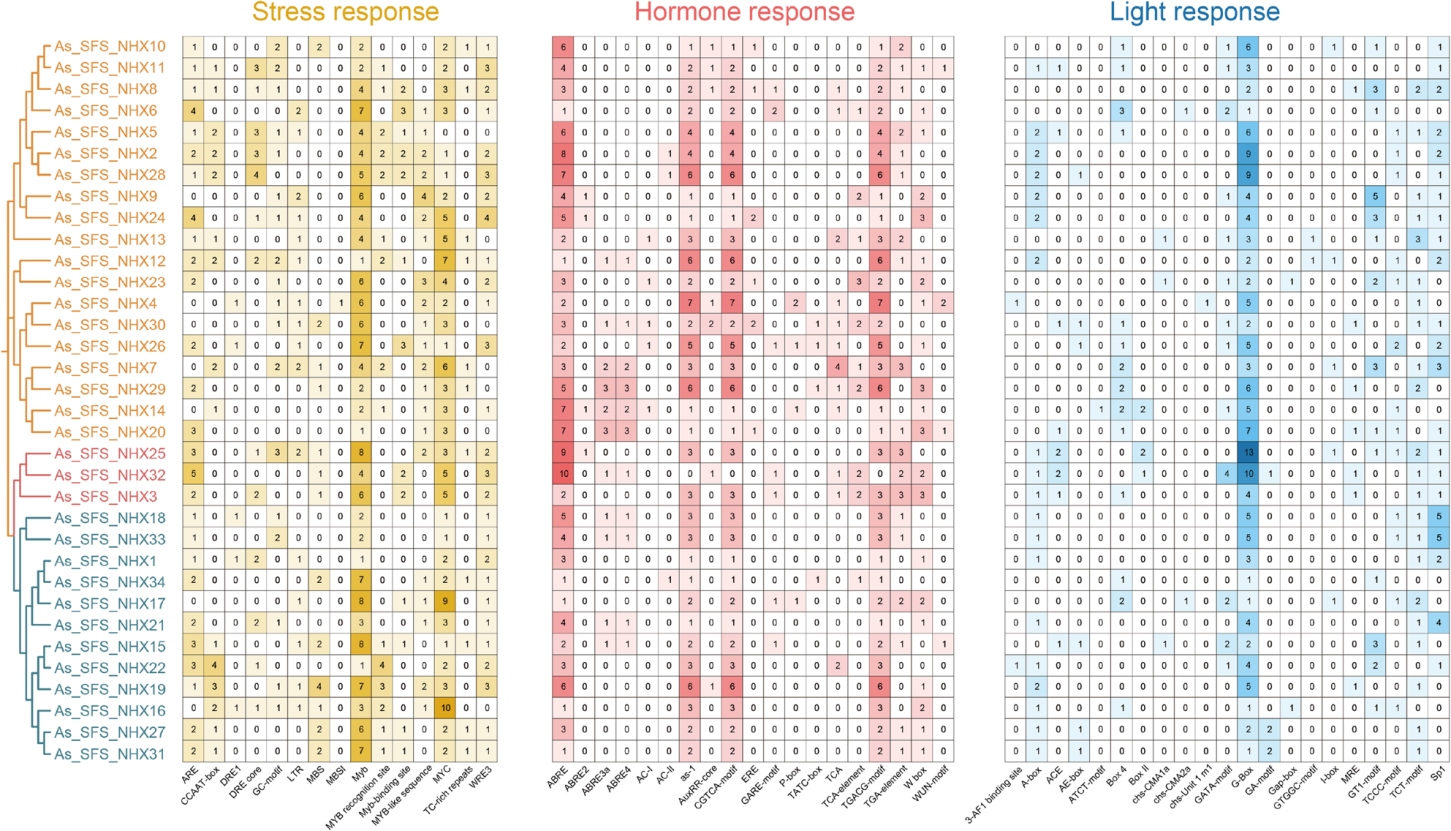
*Cis*-acting regulatory elements in the promoters of 34 NHX genes in ‘SFS’. Elements are grouped into stress-responsive, hormone-responsive, and light-responsive categories. The heatmap depicts the distribution and frequency of elements, with numbers indicating their abundance per gene

To investigate regulatory mechanisms underlying *NHX* expression, *cis*-acting elements were identified in the 2000 bp upstream promoter regions of the 34 *NHX* genes in ‘SFS’ using PlantCARE. These elements were grouped into three categories: stress-responsive (15 types), hormone-responsive (19 types), and light-responsive (21 types) (Fig. 6). A total of 577 stress-related elements were detected, associated with responses to low temperature, drought, anaerobic induction, and wounding. Hormone-related elements totaled 598, covering ABA, salicylic acid (SA), auxin (IAA), gibberellin (GA), and jasmonic acid (JA) signaling pathways. Among these, ABA-responsive elements (ABRE) were the most abundant, present in all genes, underscoring their central role in regulation. *As_SFS_NHX32* contained the highest number of hormone-responsive elements (10), suggesting a strong involvement in hormone signaling. MeJA-responsive elements were widespread, occurring in all genes except *As_SFS_NHX32*.

All *NHX* genes also contained light-responsive elements, with the G-box being the most prevalent and present in every promoter. Notably, PM-class genes *As_SFS_NHX3*, *As_SFS_NHX25*, and *As_SFS_NHX32* harbored the highest densities of stress-, hormone-, and light-responsive elements, indicating their importance in growth, development, and abiotic stress adaptation in naked oat.

### Expression profiling of oat *NHX* genes under salt stress

**Fig. 7:**
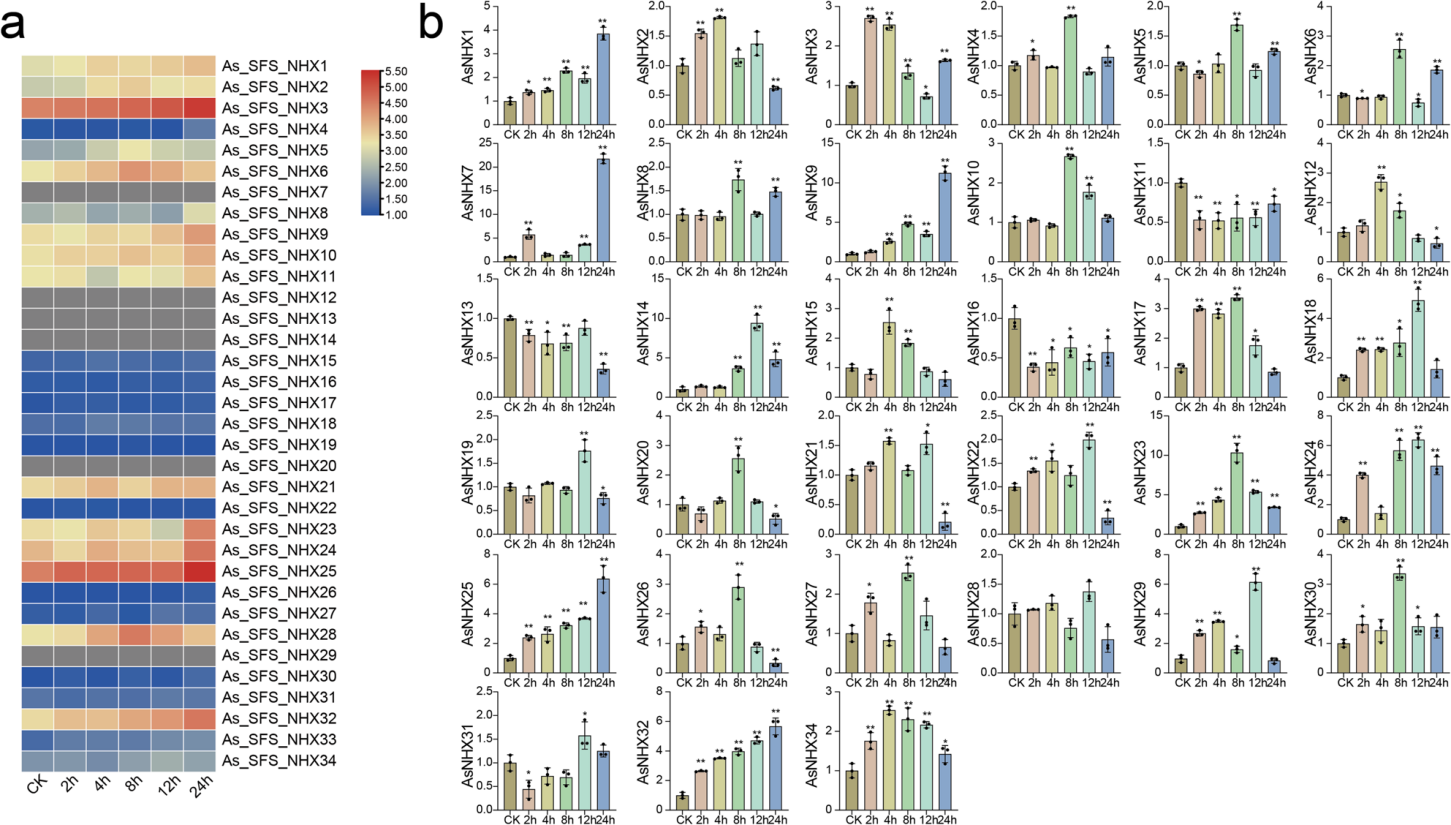
Expression patterns of 34 NHX genes under salt stress. **a** Transcriptome profiling (RNA-seq) of 34 AsNHX genes in leaf under 100 mM NaCl treatment at 0 h (CK), 2 h, 4 h, 8 h, 12 h, and 24 h. The heatmap illustrates log₂(TPM + 1) values. The color scale in both heatmaps represents expression levels, from low (blue) to high (red). **b** qRT-PCR validation of AsNHX gene expression in leaf under identical salt stress conditions. Expression levels are presented relative to the 0 h control. Statistical significance between each treatment time point and the control was determined by Student’s t-test (*P < 0.05, **P < 0.01)

Given the well-established role of *NHX* genes in salt tolerance, the expression of 34 *AsNHX* genes was assessed in naked oat exposed to NaCl for 0, 2, 4, 8, 12, and 24 h (Fig. 7a). Heatmap analysis of log₂(TPM + 1) values showed that *As_SFS_NHX7*/*12*/*13*/*14*/*20*/*29* were undetectable, while *As_SFS_NHX4*/*15/16*/*17*/*18*/*19*/*22*/*26*/*27/30*/*31*/*33*/*34* exhibited no significant changes under salt stress. In contrast, PM-class genes *As_SFS_NHX3/25/32* responded strongly, with expression levels increasing steadily and peaking at 24 h, suggesting salt-stress regulation.

To validate transcriptome findings, naked oat seedlings were treated with 100 mM NaCl, and expression of 33 *NHX* genes was analyzed by qRT-PCR at the same time points (Fig. 7b). Expression of *AsNHX33* could not be accurately quantified due to sequence identity with part of *AsNHX18*. The analysis revealed diverse expression patterns. *AsNHX11* and *AsNHX16* were significantly downregulated, while most genes were upregulated under salt treatment. Notably, *AsNHX1/7/9/14/23/24/25/32* exhibited the strongest induction. Several genes displayed time-dependent dynamics, being either early- or late-responsive. For example, *AsNHX3/12/15/17/18/23/30/34* were upregulated initially but declined later, whereas *AsNHX31* decreased first and then increased. These patterns indicate that *AsNHX* genes contribute differentially to salt stress responses, with some functioning in early signaling and others in later adaptive processes.

### Protein-Protein interaction network analysis of *NHX* genes

**Fig. 8:**
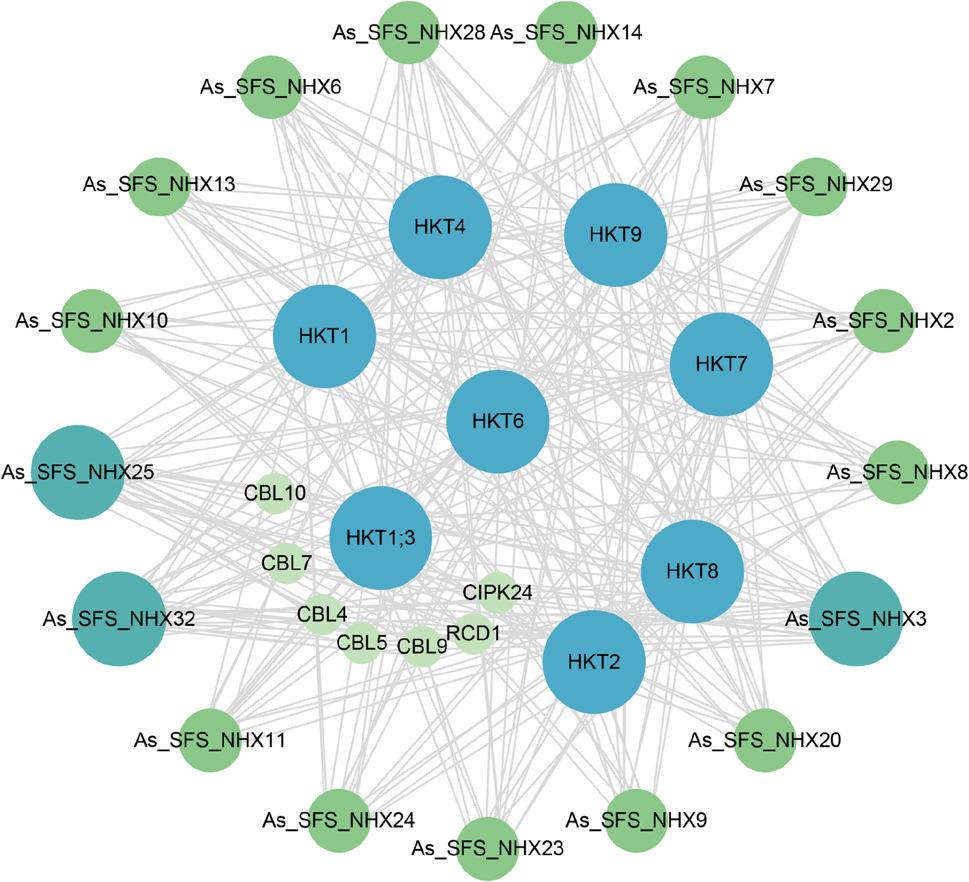
Predicted protein-protein interactions (PPIs) of NHX proteins in the ‘SFS’ genome. Gray lines denote potential interactions; node size reflects the number of interacting partners

To identify potential partners of NHX proteins in salt stress adaptation, a protein–protein interaction (PPI) network was constructed using the STRING database (Fig. 8). No direct interactions among AsNHX proteins were predicted. Of the 34 AsNHX proteins, 17 were included in the PPI network, interacting primarily with members of the HKT and CBL families. HKT proteins mediate Na⁺ and K⁺ transport, while CBL proteins regulate intracellular Na⁺ and K⁺ homeostasis, enhancing salt tolerance. These associations suggest that NHX proteins act coordinately with other salt-responsive proteins to mediate adaptation. PM-class genes *As_SFS_NHX3*, *As_SFS_NHX25*, and *As_SFS_NHX32* displayed more extensive interactions with HKT and CBL proteins, further underscoring their key role in ion regulation and salt stress response.

To validate potential interactions predicted by the PPI network, we analyzed the expression of interaction partners under the same salt stress conditions (Fig. S2). Genes including *HKT1*, *HKT2*, *HKT9*, and *CBL10* were significantly upregulated, with induction kinetics overlapping those of core *AsNHX* genes (*AsNHX3*, *AsNHX25*), supporting potential functional cooperation in the salt stress response.

### Subcellular localization of PM-class AsNHX proteins

To experimentally confirm plasma membrane localization of key AsNHX transporters, AsNHX3-GFP, AsNHX25-GFP, and AsNHX32-GFP constructs were transiently expressed in *Nicotiana benthamiana* leaves. Confocal imaging revealed fluorescence exclusively at the cell periphery, delineating the plasma membrane (Fig. S3). These results provide direct experimental evidence that AsNHX3, AsNHX25, and AsNHX32 are plasma membrane-localized, confirming their bioinformatic classification and supporting their role in Na^+^ exclusion to mitigate salt stress.

## Discussion

Na⁺/H⁺ antiporters encoded by the *NHX* gene family are critical for plant salt tolerance by maintaining intracellular pH and ion homeostasis [15]. Our genome-wide analysis identified 126 *NHX* genes across four oat cultivars, revealing both conserved features and lineage-specific innovations. Variation in gene number (30–34) and the predominance of accessory OGGs highlight substantial PAV, potentially underlying oats’ adaptability to marginal soils. Gene family expansion through duplication is a common evolutionary strategy; for instance, in allopolyploid cotton, tetraploids possess *NHX* numbers nearly equivalent to the sum of their diploid progenitors [26]. Similar *NHX* expansions occur in soybean and alfalfa, indicating that duplication is a widespread mechanism to cope with environmental stress [38, 39].

Phylogenetic classification grouped oat NHX proteins into vacuolar (Vac), endosomal (Endo), and plasma membrane (PM) classes, consistent with classifications in *Arabidopsis thaliana*, rice, *Populus trichocarpa*, *Beta vulgaris*, kiwifruit, and tea plant [27, 28, 40–43]. This conserved classification suggests that *NHX* functional specialization emerged early in angiosperm evolution and remains highly conserved. The Vac-class was the largest, a trend observed in *Beta vulgaris* and *Populus trichocarpa* [17, 42], underscoring the fundamental role of vacuolar sodium sequestration. Strong collinearity and sequence similarity between oat and wheat *NHX* genes support a close evolutionary relationship and parallel mechanisms of gene duplication and diversification [27].

Our Ka/Ks analysis further illuminates the evolutionary pressures shaping this family. Consistent with findings in cotton, tea plants, and other species, most oat *NHX* homologous pairs were under purifying selection (Ka/Ks < 1), indicating constraint on their core functions [26]. However, several pairs (e.g., *As_Marvel_NHX11/27*) exhibited Ka/Ks > 1, a hallmark of positive selection often associated with adaptive evolution. These observations align with reports of stress-responsive *NHX* genes in cotton and wheat, suggesting ongoing adaptive evolutionary pressures [26, 44]. Subgenomic mapping revealed asymmetric *NHX* distribution, with the D subgenome contributing most genes, a phenomenon also reported in other polyploids and potentially linked to subgenome dominance in stress adaptation [45–47]. The phylogenetic position of ‘SFS’ as the earliest-diverging lineage may reflect its adaptation to specific environments. As a naked oat, SFS’s distinct evolutionary history could reflect genetic changes associated with hull loss, a trait emerging early during cultivar diversification.

Structural and motif analyses revealed class-specific organization and functional divergence. Ten conserved motifs showed distinct patterns across classes: PM-class genes consistently contained motifs 1, 5, 6, 8, and 9 and carried the Crp and NhaP superfamily domains, which are important for cAMP sensing, K⁺/H⁺ exchange, and salt stress response [48–50]. Vac-class members contained all 10 motifs, reflecting higher functional complexity. The universal presence of the amiloride-binding site is a conserved feature critical for transport activity, previously documented in *Arabidopsis*, *Zanthoxylum*, and cotton NHX proteins [26, 51, 52]. Variability in intron number, particularly the elevated count in PM-class genes, represents a conserved architectural trait observed in *Populus*, soybean, and cotton [26, 38, 53]. This diversity may underlie regulatory flexibility and alternative splicing, broadening the functional repertoire of *NHX* genes under stress.

Previous studies suggest that *cis*-acting elements play a crucial role in regulating plant stress tolerance, growth, and development [54]. Promoter analysis uncovered a complex regulatory landscape. All *AsNHX* promoters carried elements for abiotic stress (e.g., cold, drought), hormone signaling (e.g., ABA, SA, JA), and light response. The universal presence of ABRE elements suggests ABA-dependent regulation, consistent with studies in rice, *Arabidopsis*, and *Brassica napus* [55, 56]. he pervasive presence of MeJA-responsive motifs indicates potential cross-talk between jasmonic acid signaling and salt tolerance, a mechanism increasingly documented in species such as cotton and tomato [26, 57, 58]. Notably, PM-class members (*As_SFS_NHX3*,* As_SFS_NHX25*,* As_SFS_NHX32*) were highly enriched in stress- and hormone-responsive elements, highlighting their role as regulatory hubs. This pattern suggests that these transporters may function as master regulators of oat salt stress responses, analogous to *NHX1* in cucumber [44].

Protein–protein interaction predictions further supported the central roles of PM-class genes. Although no direct interactions were detected among AsNHX proteins, extensive associations were identified with HKT transporters and CBL calcium sensors, which regulate ion fluxes and downstream kinases [59, 60]. In *Arabidopsis* and *Populus*, SOS1/NHX7 interacts with CBL4/SOS3 and CIPK24/SOS2 to mediate Na⁺ extrusion, whereas Vac-class NHXs cooperate with H⁺-pumps, including H⁺-ATPase and H⁺-PPase, to facilitate vacuolar Na⁺ sequestration [15, 53]. Highly connected PM-class members (As_SFS_NHX3/25/32) may act as hubs integrating Na⁺/K⁺ balance with Ca²⁺ signaling, thereby coordinating salt tolerance.

Expression profiling confirmed that most *AsNHX* genes were upregulated under salt stress, mirroring patterns in wheat and *B. napus* [12, 56]. PM-class members, particularly *AsNHX3/25/32*, exhibited strong induction with peak expression at 24 h post-treatment. In *Arabidopsis*, *NHX7/SOS1* exports Na⁺ to maintain cytosolic ion balance and a favorable Na⁺/K⁺ ratio, enhancing salt tolerance [25]. Based on promoter enrichment and predicted interactions, we propose that *AsNHX3/25/32* perform similar functions in oat. qRT-PCR validated significant upregulation of *AsNHX1/7/9/14/23/24/25/32* under salt stress. Functional orthologs, such as *TaNHX3* in wheat and *OsNHX1* in rice, and *GbNHX1/2* in cotton, have been shown to enhance salt tolerance in transgenic plants [18, 61]. Together, these findings identify strong candidates for functional studies and genetic improvement of oat salt tolerance.

In summary, this study systematically characterizes the *NHX* family in oat, revealing their evolutionary trajectories, structural divergence, *cis*-regulatory complexity, and transcriptional responses. PM-class members, in particular, emerge as central regulators of salt stress adaptation, coordinating ion transport and signaling pathways. These insights provide a theoretical basis for exploiting *NHX* genes in oat breeding and genetic improvement for salt tolerance.

## Conclusions

We identified 126 *NHX* genes across four oat cultivars, classified into Vac-, Endo-, and PM-classes, with significant PAV and evidence of gene duplication as a major driver of family expansion. The D subgenome contributed most to gene retention, and several gene pairs exhibited positive selection. Motif and promoter analyses demonstrated subfamily-specific functional divergence, with PM-class members enriched for stress- and hormone-responsive elements and carrying key domains (Crp and NhaP). Interaction predictions suggest cooperation with HKT and CBL proteins in ion homeostasis, while expression profiling confirmed strong induction of *AsNHX1/7/9/14/23/24/25/32* under salt stress. Collectively, these findings highlight the evolutionary diversification and functional specialization of the oat *NHX* family, providing a foundation for functional studies and genetic improvement of salt tolerance. 

## Supplementary Information

Supplementary Material 1. Table S1. List of 126 NHX gene family members identified in four oat genomes. Table S2. Physicochemical properties of 126 NHX genes. Table S3. Orthologous gene group (OGG) clustering of NHX genes across four oat genomes. Table S4. Ka, Ks, and Ka/Ks ratios for each NHX gene pair. Table S5. Basic information on AsNHX protein motifs.Table S6. Significantly enriched motifs in the NHX gene family. Table S7. Primers used in this study.

Supplementary Material 2. Fig. S1. Phylogenetic relationships among oat cultivars. Fig. S2. Expression patterns of potential NHX-interacting proteins under salt stress. The heatmap displays transcript levels (log₂(TPM + 1)) of genes encoding HKT and CBL family proteins, which were predicted to interact with AsNHX proteins, in leaf under 100 mM NaCl treatment at 0 h, 2 h, 4 h, 8 h, 12 h, and 24 h. Data are derived from the same RNA-seq dataset used in Figure 7a. Fig. S3. Subcellular localization of AsNHX3, AsNHX25, and AsNHX32 in Nicotiana benthamiana leaves.

## Data Availability

The data supporting this study are available at the NCBI database ( https://www.ncbi.nlm.nih.gov/ ) under accession number PRJNA355375.

## Abbreviations

NHXs: Na⁺/H⁺ antiporters
OGGs: Orthologous gene groups
CRE: Cis-regulatory element
ABA: Abscisic acid
MeJA: Methyl jasmonate
qRT-PCR: Quantitative Real-Time PCR
SOS: SALT OVERLY SENSITIVE
HMM: Hidden Markov Model
CDD: Conserved Domain Database
ML: Maximum Likelihood
Ks: Synonymous substitution rate
Ka: Non-synonymous substitution rate
PPI: Protein-protein interaction
TPM: Transcripts Per Million
PAVs: Presence/absence variations
SA: Salicylic acid
IAA: Auxin
GA: Gibberellin
