# Supplementary material for: Genome-wide identification and expression profiling of the NHX gene family in oat (Avena sativa L.)
Source: BMC Genomics. 2026 Jan 29;27:227. doi: 10.1186/s12864-026-12519-y (PMC12922412; doi:10.1186/s12864-026-12519-y)
Supplement: Supplementary file 2 — Supplementary Material 2. Fig. S1. Phylogenetic relationships among oat cultivars. Fig. S2. Expression patterns of potential NHX-interacting proteins under salt stress. The heatmap displays transcript levels (log₂(TPM + 1)) of genes encoding HKT and CBL family proteins, which were predicted to interact with AsNHX proteins, in leaf under 100 mM NaCl treatment at 0 h, 2 h, 4 h, 8 h, 12 h, and 24 h. Data are derived from the same RNA-seq dataset used in Figure 7a. Fig. S3. Subcellular localization of AsNHX3, AsNHX25, and AsNHX32 in Nicotiana benthamiana leaves. [file 12864_2026_12519_MOESM2_ESM.docx]

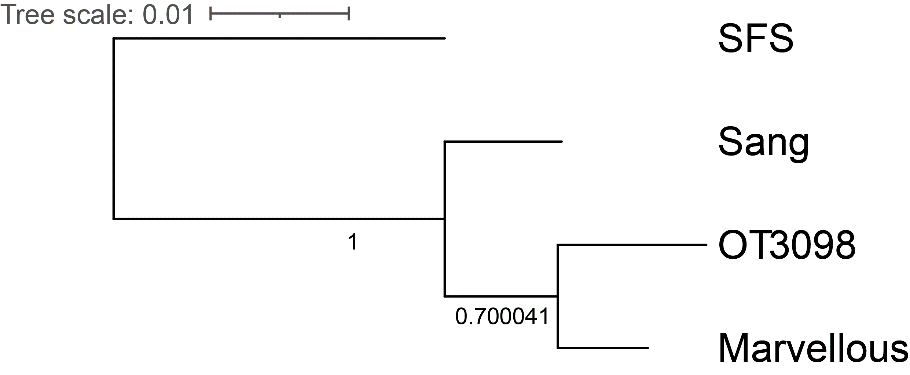


Fig. S1. Phylogenetic relationships among oat cultivars. The species tree was reconstructed using OrthoFinder. Numbers at nodes indicate bootstrap support values, and branch lengths represent genetic distances (substitutions per site).


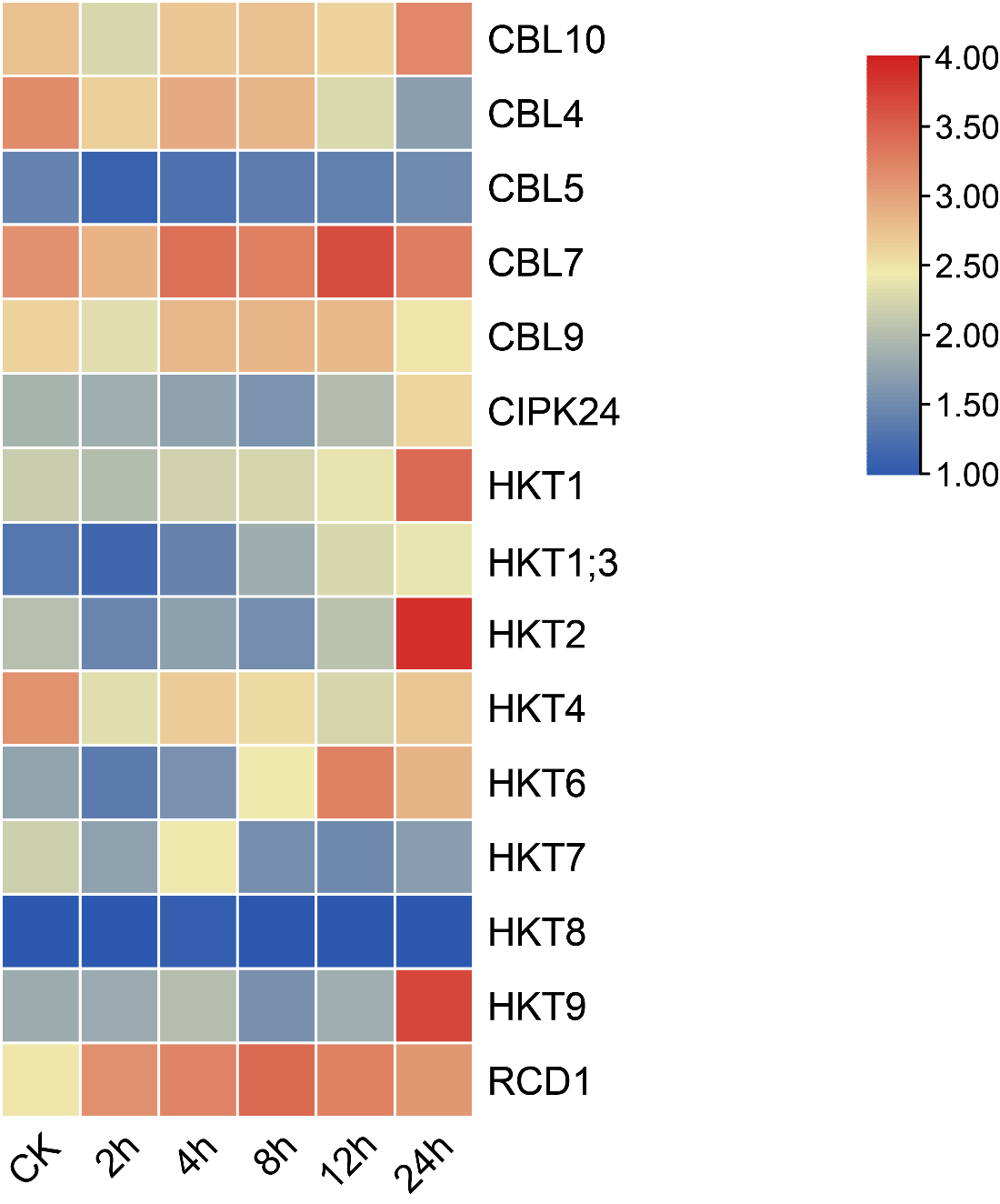


**Fig. S2. Expression patterns of potential NHX-interacting proteins under salt stress.** The heatmap displays transcript levels (log₂(TPM + 1)) of genes encoding HKT and CBL family proteins, which were predicted to interact with AsNHX proteins, in leaf under 100 mM NaCl treatment at 0 h, 2 h, 4 h, 8 h, 12 h, and 24 h. Data are derived from the same RNA-seq dataset used in Figure 7a.


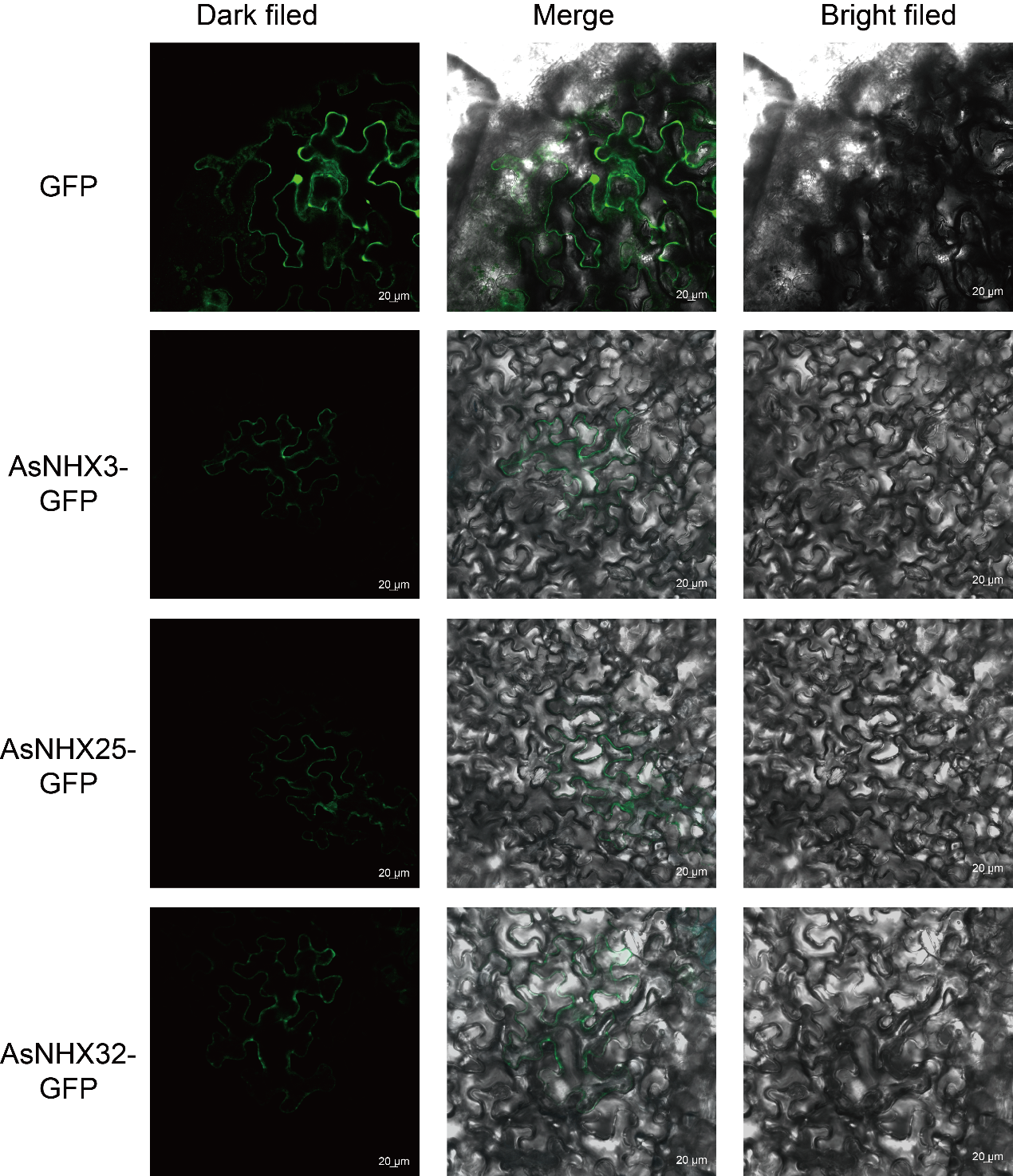


Fig. S3. Subcellular localization of AsNHX3, AsNHX25, and AsNHX32 in *Nicotiana* *benthamiana* leaves.
